# Supplementary material for: Integrated approach to model distribution and assess habitat suitability of killifish species in Oman’s local streams (wadis) under current and future climate conditions
Source: PLoS One. 2026 May 29;21(5):e0346581. doi: 10.1371/journal.pone.0346581 (PMC13221063; doi:10.1371/journal.pone.0346581)
Supplement: S1 Table — Distribution record of Aphaniops species in Oman. (DOCX) [file pone.0346581.s013.docx]

**S1 Table. Distribution record of *Aphaniops* species in Oman**

| **Family** | **Species** | **Author** | **Governorate** |
| --- | --- | --- | --- |
| Aphaniidae | *Aphaniops kruppi* | (Freyhof, Weissenbacher & Geiger, 2017) | Dhofar |
| Aphaniidae | *Aphaniops kruppi* | (Freyhof, Weissenbacher & Geiger, 2017) | Dhofar |
| Aphaniidae | *Aphaniops kruppi* | (Freyhof, Weissenbacher & Geiger, 2017) | Dhofar |
| Aphaniidae | *Aphaniops kruppi* | (Freyhof, Weissenbacher & Geiger, 2017) | Dhofar |
| Aphaniidae | *Aphaniops kruppi* | (Freyhof, Weissenbacher & Geiger, 2017) | Dhofar |
| Aphaniidae | *Aphaniops kruppi* | (Freyhof, Weissenbacher & Geiger, 2017) | Dhofar |
| Aphaniidae | *Aphaniops kruppi* | (Freyhof, Weissenbacher & Geiger, 2017) | Dhofar |
| Aphaniidae | *Aphaniops kruppi* | (Freyhof, Weissenbacher & Geiger, 2017) | Dhofar |
| Aphaniidae | *Aphaniops kruppi* | (Freyhof, Weissenbacher & Geiger, 2017) | Dhofar |
| Aphaniidae | *Aphaniops kruppi* | (Freyhof, Weissenbacher & Geiger, 2017) | Dhofar |
| Aphaniidae | *Aphaniops kruppi* | (Freyhof, Weissenbacher & Geiger, 2017) | Dhofar |
| Aphaniidae | *Aphaniops kruppi* | (Freyhof, Weissenbacher & Geiger, 2017) | Dhofar |
| Aphaniidae | *Aphaniops kruppi* | (Freyhof, Weissenbacher & Geiger, 2017) | Dhofar |
| Aphaniidae | *Aphaniops kruppi* | (Freyhof, Weissenbacher & Geiger, 2017) | Dhofar |
| Aphaniidae | *Aphaniops kruppi* | (Freyhof, Weissenbacher & Geiger, 2017) | Ash Sharqiyah South |
| Aphaniidae | *Aphaniops kruppi* | (Freyhof, Weissenbacher & Geiger, 2017) | Ash Sharqiyah North |
| Aphaniidae | *Aphaniops kruppi* | (Freyhof, Weissenbacher & Geiger, 2017) | Ash Sharqiyah North |
| Aphaniidae | *Aphaniops kruppi* | (Freyhof, Weissenbacher & Geiger, 2017) | Ash Sharqiyah North |
| Aphaniidae | *Aphaniops kruppi* | (Freyhof, Weissenbacher & Geiger, 2017) | Ash Sharqiyah North |
| Aphaniidae | *Aphaniops kruppi* | (Freyhof, Weissenbacher & Geiger, 2017) | Ash Sharqiyah North |
| Aphaniidae | *Aphaniops kruppi* | (Freyhof, Weissenbacher & Geiger, 2017) | Ash Sharqiyah North |
| Aphaniidae | *Aphaniops kruppi* | (Freyhof, Weissenbacher & Geiger, 2017) | Ash Sharqiyah North |
| Aphaniidae | *Aphaniops kruppi* | (Freyhof, Weissenbacher & Geiger, 2017) | Ash Sharqiyah North |
| Aphaniidae | *Aphaniops kruppi* | (Freyhof, Weissenbacher & Geiger, 2017) | Ash Sharqiyah North |
| Aphaniidae | *Aphaniops kruppi* | (Freyhof, Weissenbacher & Geiger, 2017) | Ash Sharqiyah North |
| Aphaniidae | *Aphaniops kruppi* | (Freyhof, Weissenbacher & Geiger, 2017) | Ash Sharqiyah North |
| Aphaniidae | *Aphaniops kruppi* | (Freyhof, Weissenbacher & Geiger, 2017) | Ash Sharqiyah North |
| Aphaniidae | *Aphaniops kruppi* | (Freyhof, Weissenbacher & Geiger, 2017) | Ash Sharqiyah North |
| Aphaniidae | *Aphaniops kruppi* | (Freyhof, Weissenbacher & Geiger, 2017) | Ash Sharqiyah North |
| Aphaniidae | *Aphaniops kruppi* | (Freyhof, Weissenbacher & Geiger, 2017) | Ash Sharqiyah North |
| Aphaniidae | *Aphaniops kruppi* | (Freyhof, Weissenbacher & Geiger, 2017) | Ash Sharqiyah North |
| Aphaniidae | *Aphaniops kruppi* | (Freyhof, Weissenbacher & Geiger, 2017) | Ash Sharqiyah North |
| Aphaniidae | *Aphaniops kruppi* | (Freyhof, Weissenbacher & Geiger, 2017) | Ad Dakhiliyah |
| Aphaniidae | *Aphaniops kruppi* | (Freyhof, Weissenbacher & Geiger, 2017) | Ad Dakhliyah |
| Aphaniidae | *Aphaniops kruppi* | (Freyhof, Weissenbacher & Geiger, 2017) | Ad Dakhiliyah |
| Aphaniidae | *Aphaniops kruppi* | (Freyhof, Weissenbacher & Geiger, 2017) | Ad Dakhliyah |
| Aphaniidae | *Aphaniops kruppi* | (Freyhof, Weissenbacher & Geiger, 2017) | Muscat |
| Aphaniidae | *Aphaniops kruppi* | (Freyhof, Weissenbacher & Geiger, 2017) | Muscat |
| Aphaniidae | *Aphaniops kruppi* | (Freyhof, Weissenbacher & Geiger, 2017) | Muscat |
| Aphaniidae | *Aphaniops kruppi* | (Freyhof, Weissenbacher & Geiger, 2017) | Muscat |
| Aphaniidae | *Aphaniops stoliczkanus* | (Day, 1872) | Dhofar |
| Aphaniidae | *Aphaniops stoliczkanus* | (Day, 1872) | Dhofar |
| Aphaniidae | *Aphaniops stoliczkanus* | (Day, 1872) | Dhofar |
| Aphaniidae | *Aphaniops stoliczkanus* | (Day, 1872) | Dhofar |
| Aphaniidae | *Aphaniops stoliczkanus* | (Day, 1872) | Dhofar |
| Aphaniidae | *Aphaniops stoliczkanus* | (Day, 1872) | Dhofar |
| Aphaniidae | *Aphaniops stoliczkanus* | (Day, 1872) | Dhofar |
| Aphaniidae | *Aphaniops stoliczkanus* | (Day, 1872) | Dhofar |
| Aphaniidae | *Aphaniops stoliczkanus* | (Day, 1872) | Al Wusta |
| Aphaniidae | *Aphaniops stoliczkanus* | (Day, 1872) | Al Wusta |
| Aphaniidae | *Aphaniops stoliczkanus* | (Day, 1872) | Ash Sharqiyah South |
| Aphaniidae | *Aphaniops stoliczkanus* | (Day, 1872) | Ash Sharqiyah South |
| Aphaniidae | *Aphaniops stoliczkanus* | (Day, 1872) | Ash Sharqiyah North |
| Aphaniidae | *Aphaniops stoliczkanus* | (Day, 1872) | Al Wusta |
| Aphaniidae | *Aphaniops stoliczkanus* | (Day, 1872) | Ash Sharqiyah South |
| Aphaniidae | *Aphaniops stoliczkanus* | (Day, 1872) | Ash Sharqiyah South |
| Aphaniidae | *Aphaniops stoliczkanus* | (Day, 1872) | Ash Sharqiyah South |
| Aphaniidae | *Aphaniops stoliczkanus* | (Day, 1872) | Ad Dakhliyah |
| Aphaniidae | *Aphaniops stoliczkanus* | (Day, 1872) | Ash Sharqiyah North |
| Aphaniidae | *Aphaniops stoliczkanus* | (Day, 1872) | Ad Dakhiliyah |
| Aphaniidae | *Aphaniops stoliczkanus* | (Day, 1872) | Ash Sharqiyah North |
| Aphaniidae | *Aphaniops stoliczkanus* | (Day, 1872) | Ash Sharqiyah North |
| Aphaniidae | *Aphaniops stoliczkanus* | (Day, 1872) | Ash Sharqiyah North |
| Aphaniidae | *Aphaniops stoliczkanus* | (Day, 1872) | Muscat |
| Aphaniidae | *Aphaniops stoliczkanus* | (Day, 1872) | Muscat |
| Aphaniidae | *Aphaniops stoliczkanus* | (Day, 1872) | Ad Dakhliyah |
| Aphaniidae | *Aphaniops stoliczkanus* | (Day, 1872) | Ad Dakhliyah |
| Aphaniidae | *Aphaniops stoliczkanus* | (Day, 1872) | Ad Dakhliyah |
| Aphaniidae | *Aphaniops stoliczkanus* | (Day, 1872) | Ad Dakhliyah |
| Aphaniidae | *Aphaniops stoliczkanus* | (Day, 1872) | Muscat |
| Aphaniidae | *Aphaniops stoliczkanus* | (Day, 1872) | Muscat |
| Aphaniidae | *Aphaniops stoliczkanus* | (Day, 1872) | Ash Sharqiyah North |
| Aphaniidae | *Aphaniops stoliczkanus* | (Day, 1872) | Ad Dakhiliyah |
| Aphaniidae | *Aphaniops stoliczkanus* | (Day, 1872) | Ad Dakhliyah |
| Aphaniidae | *Aphaniops stoliczkanus* | (Day, 1872) | Ad Dakhliyah |
| Aphaniidae | *Aphaniops stoliczkanus* | (Day, 1872) | Ad Dakhliyah |
| Aphaniidae | *Aphaniops stoliczkanus* | (Day, 1872) | Al Batinah South |
| Aphaniidae | *Aphaniops stoliczkanus* | (Day, 1872) | Ad Dakhiliyah |
| Aphaniidae | *Aphaniops stoliczkanus* | (Day, 1872) | Al Batinah South |
| Aphaniidae | *Aphaniops stoliczkanus* | (Day, 1872) | Al Batinah South |
| Aphaniidae | *Aphaniops stoliczkanus* | (Day, 1872) | Ad Dakhiliyah |
| Aphaniidae | *Aphaniops stoliczkanus* | (Day, 1872) | Muscat |
| Aphaniidae | *Aphaniops stoliczkanus* | (Day, 1872) | Al Batinah South |
| Aphaniidae | *Aphaniops stoliczkanus* | (Day, 1872) | Al Batinah South |
| Aphaniidae | *Aphaniops stoliczkanus* | (Day, 1872) | Al Batinah South |
| Aphaniidae | *Aphaniops stoliczkanus* | (Day, 1872) | Ad Dakhliyah |
| Aphaniidae | *Aphaniops stoliczkanus* | (Day, 1872) | Muscat |
| Aphaniidae | *Aphaniops stoliczkanus* | (Day, 1872) | Muscat |
| Aphaniidae | *Aphaniops stoliczkanus* | (Day, 1872) | Muscat |
| Aphaniidae | *Aphaniops stoliczkanus* | (Day, 1872) | Muscat |
| Aphaniidae | *Aphaniops stoliczkanus* | (Day, 1872) | Muscat |
| Aphaniidae | *Aphaniops stoliczkanus* | (Day, 1872) | Muscat |
| Aphaniidae | *Aphaniops stoliczkanus* | (Day, 1872) | Al Batinah South |
| Aphaniidae | *Aphaniops stoliczkanus* | (Day, 1872) | Muscat |
| Aphaniidae | *Aphaniops stoliczkanus* | (Day, 1872) | Al Dhahira |
| Aphaniidae | *Aphaniops stoliczkanus* | (Day, 1872) | Muscat |
| Aphaniidae | *Aphaniops stoliczkanus* | (Day, 1872) | Muscat |
| Aphaniidae | *Aphaniops stoliczkanus* | (Day, 1872) | Muscat |
| Aphaniidae | *Aphaniops stoliczkanus* | (Day, 1872) | Muscat |
| Aphaniidae | *Aphaniops stoliczkanus* | (Day, 1872) | Muscat |
| Aphaniidae | *Aphaniops stoliczkanus* | (Day, 1872) | Muscat |
| Aphaniidae | *Aphaniops stoliczkanus* | (Day, 1872) | Muscat |
| Aphaniidae | *Aphaniops stoliczkanus* | (Day, 1872) | Muscat |
| Aphaniidae | *Aphaniops stoliczkanus* | (Day, 1872) | Muscat |
| Aphaniidae | *Aphaniops stoliczkanus* | (Day, 1872) | Muscat |
| Aphaniidae | *Aphaniops stoliczkanus* | (Day, 1872) | Al Batinah South |
| Aphaniidae | *Aphaniops stoliczkanus* | (Day, 1872) | Al Batinah North |
| Aphaniidae | *Aphaniops stoliczkanus* | (Day, 1872) | Al Batinah North |
| Aphaniidae | *Aphaniops stoliczkanus* | (Day, 1872) | Al Batinah North |
| Aphaniidae | *Aphaniops stoliczkanus* | (Day, 1872) | Al Buraymi |
| Aphaniidae | *Aphaniops stoliczkanus* | (Day, 1872) | Al Bureimi |
| Aphaniidae | *Aphaniops stoliczkanus* | (Day, 1872) | Al Buraymi |
| Aphaniidae | *Aphaniops stoliczkanus* | (Day, 1872) | Al Batinah North |
| Aphaniidae | *Aphaniops stoliczkanus* | (Day, 1872) | Al Buraymi |
| Aphaniidae | *Aphaniops stoliczkanus* | (Day, 1872) | Al Buraymi |
| Aphaniidae | *Aphaniops stoliczkanus* | (Day, 1872) | Al Buraymi |
| Aphaniidae | *Aphaniops stoliczkanus* | (Day, 1872) | Al Batinah North |
| Aphaniidae | *Aphaniops stoliczkanus* | (Day, 1872) | Al Batinah North |
| Aphaniidae | *Aphaniops stoliczkanus* | (Day, 1872) | Al Buraymi |
| Aphaniidae | *Aphaniops stoliczkanus* | (Day, 1872) | Al Buraymi |
| Aphaniidae | *Aphaniops stoliczkanus* | (Day, 1872) | Al Batinah North |
| Aphaniidae | *Aphaniops stoliczkanus* | (Day, 1872) | Al Batinah North |
| Aphaniidae | *Aphaniops stoliczkanus* | (Day, 1872) | Al Batinah North |
| Aphaniidae | *Aphaniops stoliczkanus* | (Day, 1872) | Al Batinah North |
| Aphaniidae | *Aphaniops stoliczkanus* | (Day, 1872) | Al Batinah North |
| Aphaniidae | *Aphaniops stoliczkanus* | (Day, 1872) | Al Batinah North |
| Aphaniidae | *Aphaniops stoliczkanus* | (Day, 1872) | Al Buraymi |
| Aphaniidae | *Aphaniops stoliczkanus* | (Day, 1872) | Al Buraymi |
| Aphaniidae | *Aphaniops stoliczkanus* | (Day, 1872) | Al Buraymi |
| Aphaniidae | *Aphaniops stoliczkanus* | (Day, 1872) | Musandam |
| Aphaniidae | *Aphaniops stoliczkanus* | (Day, 1872) | Musandam |
