# Supplementary material for: Integrated approach to model distribution and assess habitat suitability of killifish species in Oman’s local streams (wadis) under current and future climate conditions
Source: PLoS One. 2026 May 29;21(5):e0346581. doi: 10.1371/journal.pone.0346581 (PMC13221063; doi:10.1371/journal.pone.0346581)
Supplement: S2 Table — Physical and environmental site characteristics in Hajar Mountain wadis, Oman. (DOCX) [file pone.0346581.s014.docx]

**S2 Table. Physical and environmental site characteristics in Hajar Mountain wadis, Oman.**

| **Site Name** | **Stream ID** | **Coordinates** | **Site Description** | **Vegetation** | **Substrate type/ Soil texture** | **Water flow/ Shade** |
| --- | --- | --- | --- | --- | --- | --- |
| **Wadi Al Amirat (1)** | **A1** | 23°29'59.16"N  58°29'38.61"E | Upstream mountain wadi site in central Muscat with preserved riparian vegetation. Experiences moderate disturbances from nearby roads and habitat fragmentation. Represents relatively pristine mountain wadi ecosystem with recreational conservation value. | (+++) High riparian vegetation | Gravel (10.56%), Silt (1.9%), Caly (3.42%), Sand (84.12%)/ Sand | Stagnant water/ partially shaded area due to heavy vegetation |
| **Wadi Al Amirat (2)** | **A2** | 23°32'24.29"N  58°30'50.97"E | Midstream site near Al-Atkia industrial area representing moderate urbanization gradient. Impacted by dam operations, sewage treatment facilities, and recreational hiking activities. Shows mixed urban-natural interface characteristics. | (+++) High riparian vegetation mostly reeds and aquatic algae | Gravel (40.04%), Silt (9.74%), Caly (5.1%), Sand (45.12%)/ Sandy Loam | Running water/ unshaded area |
| **Wadi Aday** | **A3** | 23°35'20.40"N  58°31'22.08"E | Downstream site in Wilayat Muttarh extending to Al Qurm, representing intense urban development conditions. Experiences significant disturbances from roads, electricity infrastructure, and adjacent urban settlements. | (+) Riparian vegetation and aquatic algae | Gravel (48.1%), Silt (1.45%), Caly (3%), Sand (47.45%)/ Sand | Mixed flow between stagnant and running / unshaded area |
| **Ain Wadhah (1)** | **AW1** | 22°59'28.13"N  57°17'34.55"E | Upstream mountain-edge site in Wilayat Bahla representing oligotrophic mountain-stream interface with clear mountain-agricultural transition. Located on southern Hajar Mountains with Late Tertiary alluvial deposits. Shows distinctive geological substrate and minimal tourism impacts with traditional agricultural activities visible in surrounding areas. | (+) Riparian vegetation mostly reeds | Gravel (64.73%), Silt (0.9%), Caly (1.85%), Sand (32.52%)/ Sand | Mixed flow between stagnant and running / unshaded area |
| **Ain Wadhah (2)** | **AW2** | 22°59'26.74"N  57°17'33.40"E | Midstream Mountain site with exceptional riparian vegetation and good water quality representing pristine oligotrophic conditions. Characteristic of mountain groundwater-fed systems with minimal anthropogenic influence, showing high gravel content and specialized mountain-adapted community assemblages typical of cold-water oligotrophic environments. | (++) High riparian vegetation mostly reeds | Gravel (90.49%), Silt (0.67%), Caly (0.43%), Sand (8.4%) | Mostly running water sometimes impacted with wind thus enable to measure/ partially shaded area due to heavy vegetation |
| **Ain Wadhah (3)** | **AW3** | 22°59'12.36"N  57°17'28.13"E | Downstream Mountain site with few shrub vegetation and distinctive rocky substrate formations. Maintains characteristic mountain-stream oligotrophic conditions with partial shading from large geological rock formations. Represents lower reach of mountain groundwater system with reduced vegetation but preserved water quality characteristics. | Few shrubs | Gravel (60.44%), Silt (1.14%), Caly (2.51%), Sand (35.91%)/ Sand | Mixed flow between stagnant and running/ partially shaded because of big rock formation |
| **Wadi Darsait (1)** | **D1** | 23°36'45.04"N  58°32'41.14"E | Upstream urban site representing beginning of complete wadi-to-sea continuum system in Muscat. Experiences significant disturbances from adjacent municipal buildings, highway infrastructure, and urban development. Represents urban headwaters within municipal development zone with characteristic urban stream syndrome effects and reduced natural buffering. | (++) Riparian vegetation and aquatic algae | Gravel (36.67%), Silt (3.94%), Caly (4.11%), Sand (55.27%)/ Sand | Running water/ unshaded area |
| **Wadi Darsait (2)** | **D2** | 23°37'11.79"N  58°32'23.18"E | Midstream urban site significantly impacted by major sewage treatment plant operations, water infrastructure, and road systems. Located under bridge structure representing middle reaches of urban wadi system with direct wastewater treatment influences creating unique anthropogenic environmental conditions not found in other watershed groups. | (++) Riparian vegetation and aquatic algae | Gravel (14.17%), Silt (10.02%), Caly (8.49%), Sand (67.32%)/ loamy Sand | Running water/ shaded area located under a bridge |
| **Wadi Darsait (3)** | **D3** | 23°37'53.40"N  58°32'30.57"E | Downstream coastal discharge site at Darsait Beach representing unique wadi mouth with direct marine influence and estuarine conditions. Impacted by coastal village settlements, active fishing activities, and marine connectivity. Creates distinctive marine-freshwater interface with minimal vegetation and unique hydrogeochemical conditions. | Almost no vegetation and few algae | Gravel (40.04%), Silt (0.9%), Caly (3.08%), Sand (55.98%)/ Sand | Running water/ unshaded area |
| **Wadi Surur** | **K1** | 23°22'41.79"N  58° 6'16.86"E | Upstream mixed-landscape site in Wilayat Samail with complex geological substrate including igneous, volcanic, metamorphic, and sedimentary rocks. Represents traditional settlement-agricultural interface in Ad Dakhiliyah region with moderate road impacts. Shows characteristic mixed-use landscape with sustainable agricultural activities and preserved wadi corridor connectivity. | (++) High riparian vegetation mostly reeds | Gravel (45.42%), Silt (14.26%), Caly (5.08%), Sand (35.23%)/ Sandy Loam | Mixed flow between stagnant and running/ unshaded area |
| **Wadi Fanja** | **K2** | 23°27'28.88"N  58° 6'24.57"E | Midstream site in Wilayat Bidbid representing sustainable mixed-use landscape with traditional agricultural patterns and village settlements. Impacted by water pumping infrastructure, rural roads, and village activities. Shows typical Ad Dakhiliyah wadi characteristics with organized agricultural plots and moderate anthropogenic influences balanced by natural wadi features. | (+) Riparian vegetation, few shrubs and few aquatic algae | Gravel (17.47%), Silt (1.22%), Caly (3.09%), Sand (78.22%)/ Sand | Running water/ unshaded area |
| **Wadi Al Khoud** | **K3** | 23°34'33.86"N  58° 7'5.94"E | Downstream site near Rusayl industrial area representing mixed urban-agricultural landscape with traditional settlement patterns including Alkhoud old town. Impacted by recreational camping activities, water pumping operations, and village development. Shows sustainable integration of traditional cultural landscapes with modern infrastructure development. | (++) High riparian vegetation | Gravel (12.47%), Silt (1.88%), Caly (5.03%), Sand (80.62%)/ Sand | Running water/ unshaded area |
