# Supplementary material for: Integrated approach to model distribution and assess habitat suitability of killifish species in Oman’s local streams (wadis) under current and future climate conditions
Source: PLoS One. 2026 May 29;21(5):e0346581. doi: 10.1371/journal.pone.0346581 (PMC13221063; doi:10.1371/journal.pone.0346581)
Supplement: S3 Table — Fish abundance and hydrological characteristics of Hajar Mountain wadis, Oman. (DOCX) [file pone.0346581.s015.docx]

**S3 Table. Fish abundance and hydrological characteristics of Hajar Mountain wadis, Oman.**

| **Site Name** | **Stream ID** | ***Aphaniops* spp. abundance** | **Hydrological Parameters** | | | | | | | | | | | |
| --- | --- | --- | --- | --- | --- | --- | --- | --- | --- | --- | --- | --- | --- | --- |
|  |  |  | **Depth (cm)** | **Width (m)** | **Velocity (m/s)** | **Water Temp (°C)** | **pH** | **EC (μS/cm)** | **TDS (mg/L)** | **Salinity (ppt)** | **DO (mg/L)** | **BOD (mg/L)** | **Turbidity (NTU)** |  |
| **Wadi Al Amirat (1)** | **A1** | 713 | 72.12 | 3.29 | 0.01 | 29.69 | 7.93 | 1928.19 | 974.02 | 0.97 | 5.83 | 2.84 | 1.81 |  |
| **Wadi Al Amirat (2)** | **A2** | 1971 | 34.92 | 7.95 | 0.40 | 29.59 | 7.89 | 2365.70 | 1198.71 | 1.22 | 6.96 | 3.74 | 2.06 |  |
| **Wadi Aday** | **A3** | 17708 | 44.38 | 5.34 | 0.17 | 30.42 | 8.26 | 1870.45 | 943.99 | 0.94 | 7.92 | 3.71 | 7.78 |  |
| **Ain Wadhah (1)** | **AW1** | 721 | 41.90 | 5.86 | 0.06 | 29.25 | 8.24 | 703.09 | 351.67 | 0.33 | 7.23 | 3.10 | 3.20 |  |
| **Ain Wadhah (2)** | **AW2** | 205 | 84.42 | 5.20 | 0.02 | 29.78 | 8.33 | 737.04 | 374.30 | 0.36 | 7.48 | 2.92 | 5.38 |  |
| **Ain Wadhah (3)** | **AW3** | 127 | 61.96 | 4.17 | 0.07 | 28.08 | 8.43 | 696.16 | 349.66 | 0.34 | 7.20 | 3.03 | 4.95 |  |
| **Wadi Darsait (1)** | **D1** | 3454 | 40.42 | 2.54 | 0.23 | 31.15 | 8.24 | 1646.11 | 827.95 | 0.82 | 7.64 | 3.32 | 3.59 |  |
| **Wadi Darsait (2)** | **D2** | 4598 | 36.93 | 6.27 | 0.12 | 30.07 | 8.63 | 1624.79 | 824.84 | 0.80 | 10.39 | 4.28 | 9.42 |  |
| **Wadi Darsait (3)** | **D3** | 52 | 37.53 | 6.68 | 0.09 | 31.58 | 8.64 | 1787.47 | 894.41 | 0.88 | 8.76 | 4.36 | 22.01 |  |
| **Wadi Surur** | **K1** | 4923 | 25.34 | 4.72 | 0.08 | 30.15 | 8.25 | 1984.19 | 1007.42 | 0.99 | 7.04 | 3.33 | 20.00 |  |
| **Wadi Fanja** | **K2** | 2204 | 29.50 | 9.33 | 0.15 | 29.88 | 8.24 | 2235.29 | 1131.99 | 1.12 | 7.02 | 3.56 | 17.31 |  |
| **Wadi Al Khoud** | **K3** | 2385 | 79.58 | 11.46 | 0.18 | 30.38 | 8.48 | 2609.50 | 1331.20 | 1.33 | 6.80 | 2.32 | 3.74 |  |
