# Supplementary material for: Integrated approach to model distribution and assess habitat suitability of killifish species in Oman’s local streams (wadis) under current and future climate conditions
Source: PLoS One. 2026 May 29;21(5):e0346581. doi: 10.1371/journal.pone.0346581 (PMC13221063; doi:10.1371/journal.pone.0346581)
Supplement: S4 Table — Discrimination and calibration metrics for Aphaniops species distribution models (SDMs). Calculated from independent test data. (DOCX) [file pone.0346581.s016.docx]

**S4 Table.** **Discrimination and calibration metrics for *Aphaniops* species distribution models (SDMs).** Calculated from independent test data:

| **Metric / Species** | **Default (0.5)** | **MaxSS (MaxSens+Spec)** | **MinROCdist** | **Threshold-Independent** |
| --- | --- | --- | --- | --- |
| **Threshold Value** | | | | |
| *A. kruppi* | 0.5 (fixed) | 0.167 ± 0.131 | 0.167 ± 0.131 | **-** |
| *A. stoliczkanus* | 0.5 (fixed) | 0.097 ± 0.070 | 0.105 ± 0.066 | **-** |
| **Sensitivity** | | | | |
| *A. kruppi* | 0.52 ± 0.126 | 0.973 ± 0.070 | 0.973 ± 0.070 | - |
| *A. stoliczkanus* | 0.448 ± 0.129 | 0.915 ± 0.069 | 0.904 ± 0.061 | - |
| **Specificity** | | | | |
| *A. kruppi* | 0.993 ± 0.003 | 0.942 ± 0.048 | 0.942 ± 0.048 | - |
| *A. stoliczkanus* | 0.990 ± 0.003 | 0.903 ± 0.039 | 0.912 ± 0.031 | - |
| **TSS** | | | | |
| *A. kruppi* | 0.513 ± 0.126 | 0.915 ± 0.090 | 0.915 ± 0.090 | - |
| *A. stoliczkanus* | 0.438 ± 0.128 | 0.817 ± 0.073 | 0.815 ± 0.073 | - |
| **Accuracy** | | | | |
| *A. kruppi* | 0.993 ± 0.003 | 0.942 ± 0.048 | 0.942 ± 0.048 | - |
| *A. stoliczkanus* | 0.989 ± 0.002 | 0.903 ± 0.039 | 0.912 ± 0.031 | - |
| **Balanced Accuracy** | | | | |
| *A. kruppi* | 0.756 ± 0.063 | 0.957 ± 0.045 | 0.957 ± 0.045 | - |
| *A. stoliczkanus* | 0.719 ± 0.064 | 0.909 ± 0.037 | 0.908 ± 0.037 | - |
| **Predicted Prevalence** | | | | |
| *A. kruppi* | 0.7% ± 0.3% | 5.9% ± 4.8% | 5.9% ± 4.8% | - |
| *A. stoliczkanus* | 1.1% ± 0.3% | 9.9% ± 3.9% | 9.0% ± 3.1% | - |
| **Observed Prevalence** | | | | |
| *A. kruppi* | 0.05% | 0.05% | 0.05% | - |
| *A. stoliczkanus* | 0.18% | 0.18% | 0.18% | - |
| **Threshold-Independent Metrics** | | | | |
| **Mean Test AUC (±SD)** | | | | |
| *A. kruppi* | - | - | - | 0.974 ± 0.014 |
| *A. stoliczkanus* | - | - | - | 0.950 ± 0.018 |
| **Continuous Boyce Index (CBI)** | | | | |
| *A. kruppi* | - | - | - | 0.909 ± 0.025 |
| *A. stoliczkanus* | - | - | - | 0.872 ± 0.028 |

**Footnotes:**

- All metrics calculated were based on independent test dataset (30% holdout), not training data, to prevent overestimation of performance (Fielding & Bell, 1997; Allouche et al., 2006; Araújo et al., 2019; Roberts et al., 2017).
- MaxSS and MinROCdist algorithm convergences for *A. kruppi* were identical (0.167 ± 0.131), resulting in identical performance metrics. This convergence occurs when the point of maximum sensitivity + specificity on the ROC curve is closest to perfect classification in high-performing models (AUC > 0.9) (Liu et al., 2013).
- The sample sizes were as follows: *A. kruppi* (n=40 total occurrences), *A. stoliczkanus* (n=90 total occurrences). Observed prevalence deviates from predicted prevalence by more than 10% in replicates (Wisz et al., 2008; Pearson et al., 2007).
