# Supplementary material for: Integrated approach to model distribution and assess habitat suitability of killifish species in Oman’s local streams (wadis) under current and future climate conditions
Source: PLoS One. 2026 May 29;21(5):e0346581. doi: 10.1371/journal.pone.0346581 (PMC13221063; doi:10.1371/journal.pone.0346581)
Supplement: S5 Table — Continuous Boyce Index (CBI) summary statistics for Aphaniops species distribution. (DOCX) [file pone.0346581.s017.docx]

**S5 Table. Continuous Boyce Index (CBI) summary statistics for *Aphaniops* species distribution.**

| **Species** | **Replicates** | **CBI (Mean ± SD)** | **Median CBI** | **Min CBI** | **Max CBI** | **95% Confidence Interval** | |
| --- | --- | --- | --- | --- | --- | --- | --- |
|  |  |  |  |  |  | **Lower** | **Upper** |
| *A. kruppi* | 15 | 0.909±0.025 | 0.902 | 0.870 | 0.945 | 0.874 | 0.942 |
| *A. stoliczkanus* | 15 | 0.872±0.028 | 0.883 | 0.805 | 0.907 | 0.815 | 0.904 |

CBI values range from -1 to +1, with positive values denoting models that are consistent with observed presence data. SD = Standard Deviation
