# Supplementary material for: Integrated approach to model distribution and assess habitat suitability of killifish species in Oman’s local streams (wadis) under current and future climate conditions
Source: PLoS One. 2026 May 29;21(5):e0346581. doi: 10.1371/journal.pone.0346581 (PMC13221063; doi:10.1371/journal.pone.0346581)
Supplement: S6 Table — Predictive uncertainty summary for Aphaniops species distribution models based on 15 bootstrap replicates. (DOCX) [file pone.0346581.s018.docx]

**S6 Table. Predictive uncertainty summary for *Aphaniops* species distribution models based on 15 bootstrap replicates.**

| **Species** | **Replicates** | **Mean SD** | **Median SD** | **Mean CV** | **Median CV** | **High Uncertainty Percent*** |
| --- | --- | --- | --- | --- | --- | --- |
| *A. kruppi* | 15 | 0.015 | 0.002 | 0.828 | 0.794 | 97.3 |
| *A. stoliczkanus* | 15 | 0.017 | 0.002 | 0.942 | 0.869 | 93.6 |

*% of model pixels with a coefficient of variation (CV) > 0.5, indicating high uncertainty. SD = Standard Deviation of predicted suitability; CV = Coefficient of Variation (SD/Mean).
