# Supplementary material for: Integrated approach to model distribution and assess habitat suitability of killifish species in Oman’s local streams (wadis) under current and future climate conditions
Source: PLoS One. 2026 May 29;21(5):e0346581. doi: 10.1371/journal.pone.0346581 (PMC13221063; doi:10.1371/journal.pone.0346581)
Supplement: S7 Table — Correlation between Habitat Suitability Index (HSI) and biodiversity metrics with bootstrap confidence intervals. (DOCX) [file pone.0346581.s019.docx]

**S7 Table. Correlation between Habitat Suitability Index (HSI) and biodiversity metrics with bootstrap confidence intervals.**

| **Biodiversity Metric** | **Pearson correlation (r)** | **95% Confidence Interval (Bootstrapped)** | | **P-value** |
| --- | --- | --- | --- | --- |
|  |  | **Lower** | **Upper** |  |
| **HSI vs Shannon** | -0.577 | -0.840 | 0.009 | 0.049* |
| **HSI vs Simpson** | -0.554 | -0.849 | 0.011 | 0.062 |
| **HSI vs Evenness** | -0.589 | -0.869 | -0.055 | 0.044* |

* Significant correlations (p < 0.05), Pearson correlation coefficient with two-tailed test, Confidence intervals derived from 1,000 bootstrap replicates.
