# Supplementary material for: Integrated approach to model distribution and assess habitat suitability of killifish species in Oman’s local streams (wadis) under current and future climate conditions
Source: PLoS One. 2026 May 29;21(5):e0346581. doi: 10.1371/journal.pone.0346581 (PMC13221063; doi:10.1371/journal.pone.0346581)
Supplement: S8 Table — Bootstrap estimates for Habitat Suitability Index (HSI) and biodiversity metrics. Estimates with 95% confidence intervals from 1,000 iterations across 12 sites. (DOCX) [file pone.0346581.s020.docx]

**S8 Table. Bootstrap estimates for Habitat Suitability Index (HSI) and biodiversity metrics.** Estimates with 95% confidence intervals from 1,000 iterations across 12 sites.

**S8A Table: HSI bootstrap confidence intervals by site**

| **Stream ID** | **HSI (Original)** | **Bootstrap (Mean ± SD)** | **95% Confidence Interval (Bootstrapped)** | |
| --- | --- | --- | --- | --- |
|  |  |  | **Lower** | **Upper** |
| A1 | 0.584 | 0.518 ± 0.167 | 0.096 | 0.776 |
| A2 | 0.490 | 0.443 ± 0.165 | 0.045 | 0.709 |
| A3 | 0.945 | 0.372 ± 0.162 | 0.044 | 0.664 |
| AW1 | 0.587 | 0.678 ± 0.111 | 0.407 | 0.838 |
| AW2 | 0.526 | 0.443 ± 0.224 | 0.018 | 0.759 |
| AW3 | 0.443 | 0.382 ± 0.147 | 0.061 | 0.628 |
| D1 | 0.781 | 0.467 ± 0.137 | 0.127 | 0.692 |
| D2 | 0.581 | 0.373 ± 0.176 | 0.053 | 0.680 |
| D3 | 0.496 | 0.868 ± 0.086 | 0.642 | 0.961 |
| K1 | 0.635 | 0.532 ± 0.191 | 0.037 | 0.798 |
| K2 | 0.724 | 0.624 ± 0.145 | 0.258 | 0.821 |
| K3 | 0.433 | 0.346 ± 0.158 | 0.042 | 0.637 |

**S8B Table: Biodiversity metric descriptive statistics with bootstrap estimates**

| **Biodiversity Metric** | **Mean ± SD** | **95% Confidence Interval (Bootstrapped)** | |
| --- | --- | --- | --- |
|  |  | **Lower** | **Upper** |
| **HSI vs Shannon** | 0.607 ± 0.410 | 0.377 | 0.829 |
| **HSI vs Simpson** | 0.320 ± 0.235 | 0.188 | 0.456 |
| **HSI vs Evenness** | 0.451 ± 0.296 | 0.286 | 0.607 |
