# Supplementary material for: Integrated approach to model distribution and assess habitat suitability of killifish species in Oman’s local streams (wadis) under current and future climate conditions
Source: PLoS One. 2026 May 29;21(5):e0346581. doi: 10.1371/journal.pone.0346581 (PMC13221063; doi:10.1371/journal.pone.0346581)
Supplement: S9 Table — Leave-One-Out Cross-Validation results for HSI model. (DOCX) [file pone.0346581.s021.docx]

**S9 Table. Leave-One-Out Cross-Validation results for HSI model**
**S9A Table: Site-specific LOOCV results**

| **Stream ID** | **HSI Observed** | **HSI Predicted (LOOCV)** | **Residual** | **Squared Error** |
| --- | --- | --- | --- | --- |
| A1 | 0.584 | 0.503 | 0.080 | 0.006 |
| A2 | 0.490 | 0.266 | 0.224 | 0.050 |
| A3 | 0.945 | 0.936 | 0.009 | 7.70744E-05 |
| AW1 | 0.587 | 0.476 | 0.111 | 0.012 |
| AW2 | 0.526 | 0.428 | 0.098 | 0.010 |
| AW3 | 0.443 | 0.306 | 0.137 | 0.019 |
| D1 | 0.781 | 0.715 | 0.066 | 0.004 |
| D2 | 0.581 | 0.238 | 0.343 | 0.117 |
| D3 | 0.496 | 0.414 | 0.083 | 0.007 |
| K1 | 0.635 | 0.412 | 0.223 | 0.050 |
| K2 | 0.724 | 0.667 | 0.057 | 0.003 |
| K3 | 0.433 | 0.261 | 0.172 | 0.029 |

**S9B Table: LOOCV performance summary metrics**

| **Metric** | **Value** | **Interpretation** |
| --- | --- | --- |
| **R² (Coefficient of Determination)** | 0.852 | Explained variance in new site predictions |
| **RMSE (Root Mean Square Error)** | 0.160 | Average prediction error on 0-1 suitability scale |
| **MAE (Mean Absolute Error)** | 0.133 | Average absolute prediction error |
| **MAPE (Mean Absolute Percentage Error)** | 24.63% | Average prediction error as percentage of observed value |

LOOCV performed with n=12, using each site once as test data. Prediction errors reflect model's ability to generalize to new, unseen locations.

**S9C Table: Residual analysis**

| **Statistic** | **Value** |
| --- | --- |
| **Mean residual** | 0.133 |
| **Standard deviation of residuals** | 0.094 |
| **Minimum residual** | 0.009 (site A3) |
| **Maximum residual** | 0.343 (site D2) |
| **Residuals > 0** | 12 sites (100%) |
| **Residuals < 0** | 0 sites (0%) |

Interpretation: The LOOCV model shows systematic under-prediction bias (positive mean residual = 0.133) with moderate variability (SD = 0.094). The largest errors occurred at Sites D2 (0.343) and K1 (0.223), indicating these wadis have unique ecological characteristics not captured when the model is trained on other sites.
