# Supplementary material for: Integrated approach to model distribution and assess habitat suitability of killifish species in Oman’s local streams (wadis) under current and future climate conditions
Source: PLoS One. 2026 May 29;21(5):e0346581. doi: 10.1371/journal.pone.0346581 (PMC13221063; doi:10.1371/journal.pone.0346581)
Supplement: S10 Table — Bray-Curtis dissimilarity matrix of beta diversity among Hajar Mountain stream sites. (DOCX) [file pone.0346581.s022.docx]

**S10 Table. Bray-Curtis dissimilarity matrix of beta diversity among Hajar Mountain stream sites.**

| **Stream ID** | **D1** | **D2** | **D3** | **A1** | **A2** | **A3** | **AW1** | **AW2** | **AW3** | **K1** | **K2** | **K3** |
| --- | --- | --- | --- | --- | --- | --- | --- | --- | --- | --- | --- | --- |
| **D1** | 0 | 0.143 | 0 | 0.429 | 0.429 | 0.333 | 0.714 | 0.714 | 0.714 | 0.714 | 0.714 | 0.714 |
| **D2** | 0.143 | 0 | 0.143 | 0.5 | 0.5 | 0.429 | 0.75 | 0.75 | 0.75 | 0.75 | 0.75 | 0.75 |
| **D3** | 0 | 0.143 | 0 | 0.429 | 0.429 | 0.333 | 0.714 | 0.714 | 0.714 | 0.714 | 0.714 | 0.714 |
| **A1** | 0.429 | 0.5 | 0.429 | 0 | 0 | 0.143 | 0.5 | 0.5 | 0.5 | 0.25 | 0.25 | 0.25 |
| **A2** | 0.429 | 0.5 | 0.429 | 0 | 0 | 0.143 | 0.5 | 0.5 | 0.5 | 0.25 | 0.25 | 0.25 |
| **A3** | 0.333 | 0.423 | 0.333 | 0.1429 | 0.143 | 0 | 0.714 | 0.714 | 0.714 | 0.429 | 0.429 | 0.429 |
| **AW1** | 0.714 | 0.75 | 0.714 | 0.5 | 0.5 | 0.714 | 0 | 0 | 0 | 0.5 | 0.5 | 0.5 |
| **AW2** | 0.714 | 0.75 | 0.714 | 0.5 | 0.5 | 0.714 | 0 | 0 | 0 | 0.5 | 0.5 | 0.5 |
| **AW3** | 0.714 | 0.75 | 0.714 | 0.5 | 0.5 | 0.714 | 0 | 0 | 0 | 0.5 | 0.5 | 0.5 |
| **K1** | 0.714 | 0.75 | 0.714 | 0.25 | 0.25 | 0.429 | 0.5 | 0.5 | 0.5 | 0 | 0 | 0 |
| **K2** | 0.714 | 0.75 | 0.714 | 0.25 | 0.25 | 0.429 | 0.5 | 0.5 | 0.5 | 0 | 0 | 0 |
| **K3** | 0.714 | 0.75 | 0.714 | 0.25 | 0.25 | 0.429 | 0.5 | 0.5 | 0.5 | 0 | 0 | 0 |
