# Supplementary material for: Integrated approach to model distribution and assess habitat suitability of killifish species in Oman’s local streams (wadis) under current and future climate conditions
Source: PLoS One. 2026 May 29;21(5):e0346581. doi: 10.1371/journal.pone.0346581 (PMC13221063; doi:10.1371/journal.pone.0346581)
Supplement: S11 Table — Habitat Suitability Curve (HSC) parameter contributions for Aphaniops spp. (DOCX) [file pone.0346581.s023.docx]

**S11 Table. Habitat Suitability Curve (HSC) parameter contributions for *Aphaniops* spp.**

| **Parameter** | **Mean Suitability** | **Median Suitability** | **SD Suitability** | **Min Suitability** | **Max Suitability** |
| --- | --- | --- | --- | --- | --- |
| **DO** | 0.771 | 0.902 | 0.308 | 0.039 | 0.999 |
| **Velocity** | 0.729 | 0.820 | 0.287 | 0.045 | 0.996 |
| **Width** | 0.719 | 0.809 | 0.297 | 0.102 | 0.998 |
| **Texture Numeric** | 0.719 | 0.778 | 0.247 | 0.081 | 0.994 |
| **Water Temp** | 0.707 | 0.889 | 0.354 | 0.032 | 0.999 |
| **pH** | 0.694 | 0.879 | 0.345 | 0.190 | 0.996 |
| **BOD** | 0.686 | 0.813 | 0.309 | 0.157 | 0.995 |
| **TDS** | 0.675 | 0.820 | 0.343 | 0.202 | 1.000 |
| **EC** | 0.675 | 0.821 | 0.343 | 0.200 | 0.998 |
| **Salinity** | 0.674 | 0.832 | 0.342 | 0.206 | 1.000 |
| **Depth** | 0.673 | 0.772 | 0.286 | 0.146 | 0.986 |
| **Turbidity** | 0.672 | 0.761 | 0.292 | 0.116 | 0.997 |
