# Supplementary material for: Integrated approach to model distribution and assess habitat suitability of killifish species in Oman’s local streams (wadis) under current and future climate conditions
Source: PLoS One. 2026 May 29;21(5):e0346581. doi: 10.1371/journal.pone.0346581 (PMC13221063; doi:10.1371/journal.pone.0346581)
Supplement: S12 Table — Optimal values and tolerance ranges from Aphaniops spp. Habitat Suitability Curves. (DOCX) [file pone.0346581.s024.docx]

**S12 Table. Optimal values and tolerance ranges from *Aphaniops* spp. Habitat Suitability Curves.**

| **Parameter** | | **units** | **Optimal Value (**$\mu$**)** | **SD (σ)** | **Optimal Range** | **Data Min** | **Data Max** |
| --- | --- | --- | --- | --- | --- | --- | --- |
| **Depth** | | **cm** | 47.523 | 18.818 | 28.71 - 66.34 | 20 | 90 |
| **Width** | | **m** | 6.121 | 2.496 | 3.63 - 8.62 | 2.5 | 12 |
| **Velocity** | | **m/s** | 0.141 | 0.103 | 0.04 - 0.24 | 0 | 0.45 |
| **Water Temp** | | **°C** | 30.036 | 0.745 | 29.29 - 30.78 | 28 | 32 |
| **pH** | |  | 8.282 | 0.216 | 8.07 - 8.50 | 7.8 | 8.7 |
| **EC** | | **μS/cm** | 1753.247 | 588.792 | 1164.46 - 2342.04 | 600 | 2650 |
| **TDS** | | **mg/L** | 887.236 | 300.337 | 586.90 - 1187.57 | 300 | 1350 |
| **Salinity** | | **ppt** | 0.878 | 0.307 | 0.57 - 1.18 | 0.3 | 1.35 |
| **DO** | | **mg/L** | 7.538 | 1.121 | 6.42 - 8.66 | 5 | 11 |
| **BOD** | | **mg/L** | 3.381 | 0.551 | 2.83 - 3.93 | 2 | 5 |
| **Turbidity** | | **NTU** | 8.282 | 6.618 | 1.66 - 14.9 | 1.8 | 22.1 |
| **Texture Numeric** | **Index** | | 0.697 | 0.311 | 0.39 - 1.01 | 0 | 1 |
