# Supplementary material for: Integrated approach to model distribution and assess habitat suitability of killifish species in Oman’s local streams (wadis) under current and future climate conditions
Source: PLoS One. 2026 May 29;21(5):e0346581. doi: 10.1371/journal.pone.0346581 (PMC13221063; doi:10.1371/journal.pone.0346581)
Supplement: S13 Table — Habitat Suitability Index comparison with and without substrate texture parameter. Analysis across 12 stream sites in Oman’s Hajar Mountains. (DOCX) [file pone.0346581.s025.docx]

**S13 Table. Habitat Suitability Index comparison with and without substrate texture parameter.** Analysis across 12 stream sites in Oman's Hajar Mountains.

| **Stream ID** | **HSI with Texture** | **HSI without Texture** | **HSI Difference** | **% Change** |
| --- | --- | --- | --- | --- |
| **A1** | 0.584 | 0.577 | 0.007 | 1.222 |
| **A2** | 0.490 | 0.488 | 0.002 | 0.366 |
| **A3** | 0.945 | 0.953 | -0.008 | -0.848 |
| **AW1** | 0.587 | 0.570 | 0.017 | 2.978 |
| **AW2** | 0.526 | 0.498 | 0.028 | 5.553 |
| **AW3** | 0.443 | 0.416 | 0.028 | 6.665 |
| **D1** | 0.781 | 0.765 | 0.017 | 2.212 |
| **D2** | 0.581 | 0.574 | 0.006 | 1.110 |
| **D3** | 0.496 | 0.478 | 0.018 | 3.767 |
| **K1** | 0.635 | 0.765 | -0.130 | -17.047 |
| **K2** | 0.724 | 0.734 | -0.010 | -1.367 |
| **K3** | 0.433 | 0.408 | 0.025 | 6.071 |
