# Supplementary material for: Integrated approach to model distribution and assess habitat suitability of killifish species in Oman’s local streams (wadis) under current and future climate conditions
Source: PLoS One. 2026 May 29;21(5):e0346581. doi: 10.1371/journal.pone.0346581 (PMC13221063; doi:10.1371/journal.pone.0346581)
Supplement: S14 Table — Site-specific community assembly patterns and environmental drivers in wadi systems. (DOCX) [file pone.0346581.s026.docx]

**S14 Table. Site-specific community assembly patterns and environmental drivers in wadi systems.**

| **Stream ID** | **Site Type** | **NMDS1 Range** | **Spatial Pattern** | **Key Environmental Characteristics** | **Community Assembly Drivers** |
| --- | --- | --- | --- | --- | --- |
| **AW** | Agricultural-Woodland Interface | 0.543 to 1.025 | Tight clustering in upper right quadrant | - Mountain-stream interface landscapes - Oligotrophic habitats - Warm, thermally stable waters - Low conductivity/salinity - Mountain spring waters - Site AW3: temporal change from flowing stream to isolated pools | - Strong geological and topographic environmental filtering - Mountain-influenced hydrology and geochemistry - Selective pressures favoring oligotrophic specialists - Hydrogeochemical processes from silicate/carbonate weathering |
| **K** | Traditional Wadi Systems | 0.370 to 0.661 | Tight clustering in lower right quadrant | - Mixed urban-agricultural-wadi landscape - Moderate anthropogenic influence - Arid lowland geology - Wadi geomorphology - Settled-agricultural interface impacts - Palm cultivation | - Environmental filtering from semi-arid wadi hydrology - Geological constraints - Moderate disturbance regime - Natural wadi geomorphology buffering anthropogenic stress |
| **A** | Urban Gradient Sites | -0.727 to 0.061 | Most spread across NMDS1 | - Variable urbanization gradient - A1: Moderate urbanization - A2: Fairly preserved mountain wadi - A3: Heavily developed - Habitat heterogeneity - Microenvironmental mosaics | - Complex interaction between deterministic environmental filtering and stochastic assembly - Urbanization-induced habitat heterogeneity - Urban disturbance variability overriding habitat similarities - Urban stream syndrome at varying intensities |
| **D** | Complete Urban Wadi System | -1.096 to -0.866 | Tight clustering in negative NMDS1 region | - Complete wadi-to-sea continuum - Different watershed positions: - D1: Urban headwaters - D2: Middle reaches with sewage treatment - D3: Coastal discharge with marine influence - Flashier hydrographs - Elevated nutrients - Altered channel morphology | - Regional-scale environmental filtering - Urban stream syndrome effects - Longitudinal river continuum dynamics - Disrupted longitudinal connectivity - Wastewater treatment impacts - Marine connectivity influence - Tolerant species dominance |
