# Supplementary material for: Integrated approach to model distribution and assess habitat suitability of killifish species in Oman’s local streams (wadis) under current and future climate conditions
Source: PLoS One. 2026 May 29;21(5):e0346581. doi: 10.1371/journal.pone.0346581 (PMC13221063; doi:10.1371/journal.pone.0346581)
Supplement: Attachment — Submitted filename: Response to reviewers.docx [file pone.0346581.s028.docx]

**Manuscript ID:** PONE-D-25-39513

**Title:** Integrated approach to model distribution and assess habitat suitability of killifish species in Oman's local streams (wadis) under current and future climate conditions

**Journal:** PLOS ONE

**Response to academic editor**

**Dear Dr. Daniel de Paiva Silva,**

Thank you for the opportunity to revise our manuscript and for the constructive feedback from the reviewers. We have carefully addressed all comments raised during the review process. Below, we provide detailed point-by-point responses to both the journal requirements and reviewer comments, along with descriptions of the changes made to the manuscript.

Below is a summary of the key actions taken to comply with journal requirements and strengthen the manuscript:

1. **PLOS ONE style & formatting:** The manuscript has been thoroughly reformatted to comply with all PLOS ONE style requirements, including file naming, heading hierarchy, and citation formatting. Figure and table formatting according to journal guidelines. All figures have been regenerated at 300 dpi TIFF format (600 dpi for multi-panel figures). All submitted files now follow the naming conventions: "Manuscript.docx", "Revised Manuscript with Track Changes.docx", and "Response to Reviewers.docx".
2. **Funding statements:** The complete amended funding statement and a declaration of the funders' role have been provided in the submitted cover letter.
3. **Data anonymization:** All specific geographic coordinates (longitude and latitude) have been permanently removed from the Supporting Information S1 Table to protect sensitive location data of endemic species and comply with data sharing policies.
4. **Figure copyright compliance:** All map/satellite imagery has been verified for copyright compliance. Figures 3 and 4 **(now Figs 2 and 3)** were revised by replacing Esri basemaps with public-domain vector maps from Natural Earth. We have provided a detailed Content Permission letter (submitted as "Other" file) documenting the copyright status of all figures.
5. **Citation of recommended works:** We confirm that the reviewers' comments did not include any recommendations to cite specific previously published works. However, we have added several methodologically important references in response to reviewers' requests for clarification of our analytical approaches **(see detailed responses to Reviewer #1 and #2 below)**. All new citations were independently evaluated for relevance and appropriateness before inclusion.
6. **Substantive revisions:** In response to the reviewers’ insightful comments, we have made significant enhancements to the manuscript’s scientific rigor and clarity. Key improvements include:

- **Methodological transparency:** Detailed descriptions of species distribution model calibration, accessible area (M) definition, background point selection, collinearity screening, and spatial filtering have been added.
- **Robust validation:** We expanded model evaluation to include calibration metrics (Continuous Boyce Index) and a comprehensive uncertainty framework, including a Multivariate Environmental Similarity Surface (MESS) analysis to assess extrapolation risk in future projections.
- **Quantified conservation framework:** A major new conservation gap analysis was conducted, with results consolidated in a new table **(Table 5)**. This provides clear, quantified targets against national (Oman Vision 2040) and global (CBD 30x30) benchmarks, identifying specific protection shortfalls and priority actions.
- **Improved readability:** The discussion was restructured, long paragraphs were shortened, and interpretive statements were removed from the results. Four secondary figures were moved to Supporting Information to streamline the main narrative.

We are confident that these comprehensive revisions have fully addressed all editorial and reviewer concerns. The manuscript is now substantially stronger in methodological rigor, analytical depth, and conservation applicability.

Thank you for your consideration.

Sincerely,

**Aziza S. Al Adhoobi, Ph.D. Candidate**
Department of Marine Science and Fisheries, College of Agricultural & Marine Sciences, Sultan Qaboos University, Muscat, Oman

Department of Nature Reserves, Directorate General of Biodiversity and Nature Reserves, Environment Authority, Muscat, Oman

Email: [s33756@student.squ.edu.om](mailto:s33756@student.squ.edu.om), azizco83@gmail.com

ORCID: <https://orcid.org/0000-0002-7326-8011>

**On behalf of all co-authors:**

- Saud M. Al Jufaili, Sultan Qaboos University
- Amna Al Ruheili, Sultan Qaboos University
- Wenresti Gallardo, Sultan Qaboos University

**Response to reviewers**

**Dear Reviewers,**

Thank you for your valuable feedback, which has significantly strengthened our manuscript. We have carefully addressed all comments. Below is a point-by-point summary of the key changes made.

**Response to reviewer #1:**

**Reviewer #1:** The manuscript addresses an important and timely topic by forecasting habitat suitability for two arid-land killifishes (*Aphaniops kruppi* and *A. stoliczkanus*) and linking these results to conservation planning for Omani wadis. The integration of SDMs, habitat suitability indices, community data, and protected-area overlays is promising and highlights the potential for valuable contributions to conservation science. **To further strengthen the study, I suggest the following points of improvement:**

**Author Response:** We sincerely thank the reviewer for recognizing the value and integrative nature of our study. We have carefully addressed all suggestions to strengthen the manuscript's methodological rigor, transparency, and conservation applicability. We have carefully addressed all suggestions.

**Reviewer comment #1.1**: Model calibration – Provide more detail on how the accessible area (M) was defined, how background points were selected, and whether any bias correction or model tuning procedures were applied.

**Author Response:** We apologize for the insufficient methodological detail in our original submission. We have substantially revised Methods Sections 2.1.2 and 2.1.4 to include:

**Key additions:**

- Definition of accessible area (M) using FEOW ecoregions (IDs 443 & 439).
- Rationale for 10,000 background points restricted to M.
- Application of 1 km spatial filtering to reduce autocorrelation.
- Description of model tuning settings (regularization, bootstrap replicates).

**Changes made:**

**Methods Section 2.1.2 "Species occurrence data":**

- **Lines 245-251:** "To reduce spatial autocorrelation (clustering bias) and balance data quality with sample size, we applied 1 km spatial filtering, ..."
- **Lines 253-256:** "Due to the low number of occurrences of *A. kruppi*, we decided against using spatial blocking for model evaluation to ensure sufficient data for cross-validation."
- **Lines 257-261:** "We determined both species' accessible area (M) by the FEOW ecoregion [59] for the ID 443 and 439, which represent the geographical area ..."

**Methods Section 2.1.4 "Species distribution modeling with MaxEnt":**

- **Lines 326-332:** "We used MaxEnt default settings, to generate 10,000 random background data points that are spatially limited to the entire defined accessible area (M) where a species can potentially be present and utilize environmental resources available at each location... A 10,000-point background sample was sufficient for describing all potential environmental characteristics on M, while remaining computationally efficient [95]."

**Reviewer comment #1.2**: In addition to discrimination metrics (AUC, TSS), consider reporting calibration metrics (e.g., Boyce, CBI) and briefly discuss limitations related to prevalence mismatches. Clarify the rationale for using equal suitability classes and how thresholding decisions affect interpretation.

**Author Response:** Thank you for this important suggestion. We have added calibration metrics and expanded our discussion of limitations:

**Changes made:**

**Methods Section 2.1.5 "Model validation":** Added Boyce Index procedure.

- **Lines 382-397:** "In addition to the threshold dependent metrics mentioned above, we examined the model's calibration using an assessment approach consistent with the Continuous Boyce Index (CBI) to evaluate models' calibration... This method calculates a normalized performance ratio, defined as (mean presence/mean background -- 1)/ (mean presence/mean background + 1)..."

**Results Section 3.1.1 "Species distribution model performance"**:

- **Lines 555–561**: "The CBI results showed that the models performed exceptionally well for both species: with values of 0.909 ± 0.025 for *A. kruppi* and 0.872 ± 0.028 for *A. stoliczkanus* (S4 Table)."

**Prevalence mismatches:** Discussed in Section 3.1.1 (**lines 552–554**), Section 4.1 (**lines 847-855**), and Section 4.4 (lines **1224–1230**).

**Threshold rationale:** Explained in Methods Section 2.1.5 (**lines 372–375**), and Section 4.1 (**lines 919-925**).

**Reviewer comment #1.3**: Clarify how predictors were screened for collinearity (e.g., correlation thresholds, VIF), and provide more detail about the source and processing of the elevation data.

**Author Response:** We thank the reviewer for this important methodological clarification request. We revised Section 2.1.3 to clarify:

**Collinearity screening:** “We applied a Pearson Correlation Matrix (threshold: |r| > 0.7). We selected pairwise correlation over Variance Inflation Factor (VIF)…” (**lines 282–294**)

**DEM processing:** Details on DEM source and derived metrics (**lines 295–301**).

**Changes made:** Section 2.1.3 (**lines 282–301**) comprehensively documents these protocols.

**Reviewer comment #1.4**: Acknowledge the relatively small sample size for *A. kruppi* and specify whether spatial blocking or other methods were used to reduce spatial autocorrelation during model evaluation.

**Author Response:** We have explicitly acknowledged the sample size limitation for *A. kruppi* and clarified our spatial filtering approach. The 1km spatial filtering was implemented to reduce spatial autocorrelation, and we have explained why spatial blocking was not feasible given the sample size constraints inherent to studying endemic species.

**Changes made:**

- **Methods Section 2.1.2 (lines 245–252):** Enhanced description of spatial filtering.
- **Discussion Section 4.1 (lines 999–1004):** Added paragraph on sample size implications.

**Reviewer comment #1.5**: Indicate which climate models (ensemble or single GCMs) were used for future scenarios, and include uncertainty and extrapolation maps to strengthen interpretation.

**Author Response:** We thank the reviewer for this important clarification. We specified the use of and added a comprehensive uncertainty framework:

To comprehensively address uncertainty concerns, we implemented a three-pronged approach:

- Bootstrap uncertainty quantification across 15 model replicates to assess model stability
- Multivariate Environmental Similarity Surface (MESS) analysis to identify extrapolation risk areas (new Section 2.1.6)
- Comprehensive model evaluation using independent test data and multiple validation metrics

**Changes made:**

- **GFDL-ESM4 GCM:** Section 2.1.3 (**lines 269-281**)
- **MESS analysis:** New Section 2.1.6 (**lines 399–413**)
- **Results:** New Section 3.1.4 (**lines 626–644**)
- **Discussion:** Section 4.1.3 (**lines 921–935**) discuss extrapolation risk.
- **Supporting materials:** Table 3, S4–S6 Figs, and S6 Table.

**Reviewer comment #1.6**: Present measures of uncertainty (e.g., confidence intervals) for the HSI and diversity analyses to provide a clearer picture of variability.

**Author Response:** We thank the reviewer for this important suggestion. We have incorporated comprehensive uncertainty measures through bootstrap resampling (1,000 iterations), generating 95% confidence intervals for all correlation coefficients between HSI and biodiversity metrics.

**Changes made:**

- **Methods Section 2.2.2 (lines 487-490):** "To address potential overfitting, and quantify uncertainty, we utilized bootstrap resampling (1000 iterations), to create 95 % confidence intervals for correlation coefficients and Leave-One-Out Cross-Validation (LOOCV)… "
- **Results Section 3.2.2 (lines 663-679)**
- **Supporting materials:** Table 4, Fig 6, S Tables 7-9, and S Figs 7-10

**Reviewer comment #1.7**: Strengthen the conservation section by quantifying representation within protected areas and suggesting clear targets (e.g., percentage of suitable habitat to be protected).

**Author Response:** We sincerely thank the reviewer for their insightful and constructive comments, which have significantly strengthened our manuscript. We have conducted a comprehensive conservation gap analysis and revised the manuscript accordingly.

**Changes made:**

- **Methods Sections 2.3.1 and 2.3.2 (lines 505-531)**
- **Result Section 3.3 (lines 792-820)**
- **Discussion Section 4.3:** Section 4.3.1 (**lines 1180-1191**), Section 4.3.2 (**lines 1192-1200**), and Section 4.3.3 (**lines 1201-1218**).
- **Supporting materials:** New Table 5, Figs 11 and 12, and S Figs 11 and 12.

**Reviewer comment #1.8**: Improve figure readability by adding scale bars, north arrows, clear units, and consistent palettes. Label SSPs and time periods directly on the figures.

**Author Response:** We have revised all figures accordingly. All figures have been regenerated at 300 dpi TIFF format (600 dpi for multi-panel figures) for publication quality and uploaded to the Preflight Analysis and Conversion Engine (PACE) digital diagnostic tool to ensure that figures meet PLOS requirements and were checked in NAAS before upload.

**Changes made:** All figures revised with improved readability elements; figure files resubmitted.

****************

**Response to reviewer #2:**

**Reviewer #2:** The study addresses interesting and important questions about spatial distribution patterns of species at broader scales across integrated climate change scenarios and site-based habitat assessments, raising concerns for conservation planning. However, there are some points in the text that need refinement.

**Author Response:** We sincerely thank the reviewer for the positive assessment and constructive feedback. We have carefully revised the manuscript to address all concerns regarding text organization, figure presentation, and methodological clarity.

**Reviewer comment #2.1**: There are some points in the text that need refinement because I got lost with the number of figures presented and some long paragraphs (mainly in the discussion). There are also points where the discussion mixes with the results, making the text repetitive.

**Author Response:** We have substantially revised the manuscript to improve clarity and readability:

**Changes made:**

- Discussion Sections 4.1–4.3 (**lines 838–1218**): Shorter paragraphs, clearer organization.
- Added transitional phrases throughout Discussion for improved coherence

**Reviewer comment #2.2**: Fig 1. Systematic methodology framework: I did not find it cited in the text.

**Author Response:** Thank you for catching this omission. Figure 1 is now cited in:

- Introduction (**line 172**): “Our research follows a comprehensive systematic workflow and has three interrelated aims (Fig 1).”
- Methods overview (**line 192**): “This study follows a comprehensive systematic workflow, illustrated in Fig 1.”

**Reviewer comment #2.3**: My suggestion is to include them as Supporting Information. The environmental variable and model performance plots are interesting but, in my opinion, highlight information that is not the main result.

**Author Response:** Thank you for this excellent suggestion. We have moved these figures to Supporting Information to streamline the main text and focus on primary conservation findings:

- Figure 2 (Digital elevation, temperature, precipitation maps) → Supplementary S1 Fig
- Figure 5 (ROC curves and threshold comparison) → Supplementary S2 Fig
- Figure 6 (Predictor variable contributions) → Supplementary S3 Fig
- Figure 9 (HSI model performance metrics) → Supplementary S7 Fig

**Reviewer comment #2.4**: Is it essential to have two separate tables? I would suggest just one table for both species.

**Author Response:** Thank you for this suggestion. We have merged Tables 2 and 3 into a single comprehensive table (new Table 2) that facilitates direct comparison of habitat projections for both species across all scenarios and time periods.

**Reviewer comment #2.5**: It is necessary to state what the acronym UAE means.

**Author Response:** Thank you for this catch. We have corrected **line 210** to read:

" The Oman Mountain ecoregion with the designation ID 443 stretches over the southeastern Arabian Peninsula including Oman and United Arab Emirates (UAE)."

**Reviewer comment #2.6**: The GBIF search must be referenced. When performing a search, GBIF creates a citation; thus, I suggest you include it in the text.

**Author Response:** Thank you for this important reminder. Full GBIF citations with DOIs added (lines 220–221 and References 62–66).

The full reference has been added to the reference list:

- 62. GBIF.org. GBIF Occurrence Download [Internet]. 2023 [cited 2023 Feb 15]. Available from: <https://doi.org/10.15468/nvuhqv>
- 63.	GBIF.org. GBIF Occurrence Download [Internet]. 2023 [cited 2023 Feb 15]. Available from: <https://doi.org/10.15468/ndxeqh>
- 64.	GBIF.org. GBIF Occurrence Download [Internet]. 2023 [cited 2023 Feb 15]. Available from: <https://doi.org/10.15468/sbmztx>
- 65.	GBIF.org. GBIF Occurrence Download [Internet]. 2023 [cited 2023 Feb 15]. Available from: <https://doi.org/10.11646/zootaxa.4338.3.10>
- 66.	GBIF.org. GBIF Occurrence Download [Internet]. 2023 [cited 2023 Feb 15]. Available from: <https://doi.org/10.15468/mqbnga>

**Reviewer comment #2.7**: This section does not clearly state the use of training data (used to calibrate the distribution models) and test data (used to evaluate predictive ability), nor the proportion of each relative to the total occurrences of the two species. I believe this is relevant information for fully understanding the modeling process and for the experiment's replicability.

**Author Response:** Thank you for identifying this critical gap in our methodology description. We have completely rewritten Methods **Section 2.1.4** to clarify our training/test data procedures.

“We ran the model with 500 iterations per replicate, splitting the occurrence data into training (70%) and testing (30%) subsets…” (**lines 340–344**)

**Reviewer comment #2.8**: The authors state that they used the MaxSS from the training data. Evaluating the model on the training data is similar to taking a test with the answer key, as it measures the model's fit to the data it has already seen. Generally, this leads to an overestimation of performance. The model can become overly complex, capturing not only the ecological effect but also noise and specific errors from the training set, which can lead to overfitting, making the model validation biased. Therefore, it is imperative to review the model evaluation metrics, as performance should be assessed using a test dataset. In the case of small datasets (few occurrences), cross-validation is the most recommended technique. Always prioritize evaluation with test data (or via cross-validation) to make decisions about the quality and usefulness of your species distribution model. Evaluation on training data is useful for diagnosing problems like overfitting but should not be used as the final performance measure.

**Author Response:** Thank you for this extremely important observation. We sincerely apologize for the confusion in our original manuscript. We have substantially revised our Methods and Results sections to clarify that ALL performance metrics reported in our study were calculated using independent TEST data, not training data.

**Changes made:**

- **Methods:** Section 2.1.4 (**lines 324-344**), Section 2.1.6 (**lines 412-413**)
- **Results:** Section 3.1.1 (**lines 538-543**)
- **Discussion:** Section 4.1 (**lines 856-868**).

**Reviewer comment #2.9**: If possible, this procedure could also be adopted for the "Habitat Suitability Index (HSI) model for *Aphaniops* spp."

**Author Response:** We thank the reviewer for this constructive suggestion. We implemented Leave-One-Out Cross-Validation (LOOCV) for the HSI model.

**Changes made:**

- Methods Section 2.2.2 (**lines 487-490**)
- Results Section 3.2.1 (**lines 674-679**): LOOCV results (R² = 0.003, RMSE = 0.276).
- **Supporting materials:** S9 Table and S10 Fig.

**Summary of Major Revisions:**

1. **Methodological rigor:** Added detailed calibration, uncertainty (MESS), and validation procedures.
2. **New analyses:** Continuous Boyce Index, bootstrap CIs, LOOCV, and comprehensive conservation gap analysis.
3. **Improved clarity:** Restructured text, merged tables, moved secondary figures to supplement.
4. **Compliance:** Met all journal requirements.

In addition to the specific point-by-point revisions, we have updated three key sections to reflect the enhanced analyses and ensure the manuscript's narrative accurately represents the improved study:

1. **Abstract:** Updated to include key new findings and metrics, such as:

- The quantified conservation gaps (0.31–1.34% protection of high-suitability habitat, requiring 6–24-fold increases).
- The high extrapolation risk (85-95%).
- The negative correlation between habitat suitability and biodiversity (Shannon diversity: r = -0.577).

2. **Discussion – Limitations Section (Section 4.4, lines 1220–1292):** Substantially expanded to transparently address the methodological constraints identified during review, including:

- Sample size limitations for *A. kruppi* and its implications for model thresholds.
- Predictive limitations of the Habitat Suitability Index model identified via LOOCV.
- Uncertainty associated with using a single GCM and the high extrapolation risk revealed by MESS analysis.
- Conservation gap analysis constraints (e.g., uniform management assumption, exclusion of informal protections).

3. **Conclusions (Section 5, lines 1294–1355):** Revised to synthesize the updated, evidence-based conservation recommendations, including:

We believe these revisions have thoroughly addressed all reviewer concerns, significantly strengthened the methodological rigor and narrative coherence of the manuscript, and ensured that its framing, interpretation, and conclusions are fully aligned with the enhanced analyses and updated results. We are confident that the revised manuscript now meets the high standards for publication in PLOS ONE.

**Aziza S. Al Adhoobi, Ph.D. Candidate**
Department of Marine Science and Fisheries, College of Agricultural & Marine Sciences, Sultan Qaboos University, Muscat, Oman

Department of Nature Reserves, Directorate General of Biodiversity and Nature Reserves, Environment Authority, Muscat, Oman

Email: [s33756@student.squ.edu.om](mailto:s33756@student.squ.edu.om), azizco83@gmail.com

ORCID: <https://orcid.org/0000-0002-7326-8011>

**On behalf of all co-authors:**

- Saud M. Al Jufaili, Sultan Qaboos University
- Amna Al Ruheili, Sultan Qaboos University
- Wenresti Gallardo, Sultan Qaboos University
